# Invasive Salmonellosis in Kilifi, Kenya

**DOI:** 10.1093/cid/civ737

**Published:** 2015-10-07

**Authors:** Esther Muthumbi, Susan C. Morpeth, Michael Ooko, Alfred Mwanzu, Salim Mwarumba, Neema Mturi, Anthony O. Etyang, James A. Berkley, Thomas N. Williams, Samuel Kariuki, J. Anthony G. Scott

**Affiliations:** 1 Kenya Medical Research Institute–Wellcome Trust Research Programme, Kilifi; 2 Department of Infectious Disease Epidemiology, London School of Hygiene and Tropical Medicine; 3 Nuffield Department of Clinical Medicine, Oxford University, United Kingdom; 4 Centre for Microbiological Research, Kenya Medical Research Institute, Nairobi

**Keywords:** *Salmonella*, nontyphoidal, Typhi, incidence, Kenya

## Abstract

***Background.*** Invasive salmonelloses are a major cause of morbidity and mortality in Africa, but the incidence and case fatality of each disease vary markedly by region. We aimed to describe the incidence, clinical characteristics, and antimicrobial susceptibility patterns of invasive salmonelloses among children and adults in Kilifi, Kenya.

***Methods.*** We analyzed integrated clinical and laboratory records for patients presenting to the Kilifi County Hospital between 1998 and 2014. We calculated incidence, and summarized clinical features and multidrug resistance.

***Results.*** Nontyphoidal *Salmonella* (NTS) accounted for 10.8% and 5.8% of bacteremia cases in children and adults, respectively, while *Salmonella* Typhi accounted for 0.5% and 2.1%, respectively. Among 351 NTS isolates serotyped, 160 (45.6%) were *Salmonella* Enteritidis and 152 (43.3%) were *Salmonella* Typhimurium. The incidence of NTS in children aged <5 years was 36.6 per 100 000 person-years, being highest in infants aged <7 days (174/100 000 person-years). The overall incidence of NTS in children varied markedly by location and declined significantly during the study period; the pattern of dominance of the NTS serotypes also shifted from *Salmonella* Enteritidis to *Salmonella* Typhimurium. Risk factors for invasive NTS disease were human immunodeficiency virus infection, malaria, and malnutrition; the case fatality ratio was 22.1% (71/321) in children aged <5 years and 36.7% (11/30) in adults. Multidrug resistance was present in 23.9% (84/351) of NTS isolates and 46.2% (12/26) of *Salmonella* Typhi isolates.

***Conclusions.*** In Kilifi, the incidence of invasive NTS was high, especially among newborn infants, but typhoid fever was uncommon. NTS remains an important cause of bacteremia in children <5 years of age.

Estimates of the burden of invasive *Salmonella* infections in sub-Saharan Africa are limited by the scarcity of regional data. The global burden of typhoid fever was estimated at 26.9 million cases in 2010 [1], but only Egypt and South Africa contributed to this estimate for the African continent. Within Africa, surveillance from Kenya, Tanzania, Malawi, and South Africa has shown marked regional variation in the incidence and age-specific patterns of typhoid fever [2–4]. Typhoid fever is caused by *Salmonella enterica* serovar Typhi, whereas nontyphoidal *Salmonella* (NTS) disease refers to infections caused by *Salmonella enterica* serovars other than Typhi and Paratyphi A.

The burden of NTS disease in Africa is even less well understood. In high-income countries, NTS causes a self-limiting gastroenteritis and is transmitted though contaminated food. In contrast, infection with NTS in Africa is usually invasive, causing severe life-threatening sepsis. In fact, NTS is a common cause of bacteremia in both children and adults [5, 6]. In a study of community-acquired bloodstream infections across Africa, NTS was the most common pathogenic isolate in adults; in children, it was the second most common, after *Streptococcus pneumoniae* [6]. Risk factors for invasive NTS (iNTS) disease include malaria infection, human immunodeficiency virus (HIV), and malnutrition [5, 7], all of which have a high prevalence in Africa. Existing estimates of the incidence of iNTS are derived mainly from children and high-risk groups [5, 8–10]. Population-based studies are few and thus incidence estimates are extremely limited. Modeled estimates of iNTS infection, based on extrapolations from these studies, adjusting for the effects of HIV and malaria, suggest an incidence in Africa of 227 cases per 100 000 per year across all age groups, with a case fatality rate of 20% [11].

Although incidence estimates are scarce, NTS repeatedly rank near the top in series of invasive pathogenic bacteria on the continent, suggesting that they are a cause of considerable morbidity and mortality. Vaccines are available to protect against *Salmonella* Typhi infections and are in development to prevent NTS. The case for vaccine development, and the proper evaluation and deployment of such vaccines in Africa, depends upon a sound understanding of the epidemiology of the disease in different parts of the continent. This study describes the incidence, clinical characteristics, and antimicrobial susceptibility patterns of invasive salmonellosis among children and adults admitted to Kilifi County Hospital in Coastal Kenya.

## METHODS

This is a retrospective analysis of integrated demographic, clinical, and microbiological surveillance data accumulated at the Kenya Medical Research Institute (KEMRI)–Wellcome Trust Research Programme over a period of 16 years from August 1998 through December 2014 for children ≤14 years of age, and from January 2007 through December 2014 for adults >14 years of age.

### Study Population

The study was undertaken in Kilifi County, a rural area 3 degrees south of the equator on the Indian Ocean coast, typical of much of tropical sub-Saharan Africa. Kilifi County Hospital (KCH) is located at the center of the Kilifi Health and Demographic Surveillance System (KHDSS), which was established in 2000 to monitor births, deaths, in-migration, and out-migration in a population of approximately 280 000 over an area of 891 km^2^ [12]. Events are monitored at KCH and through routine home visits every 4 months.

Since August 1998, all children (≤14 years of age) admitted to KCH, except those admitted for elective procedures or observations after minor accidents, have been investigated with blood cultures routinely on admission [5]. Full blood counts and microscopy for malaria parasites were also analyzed routinely. Clinical and laboratory data have been systematically recorded on a standard pro forma [13]. Treatment was offered according to local protocols and current World Health Organization recommendations [14].

Meningitis was suspected where 1 or more of the following signs were present: stiff neck, bulging fontanelle, lethargy, loss of consciousness, prostration or history of convulsions (except febrile convulsions), and signs of sepsis in neonates. Lumbar punctures were performed for these cases. Malnutrition was defined as a weight-for-age *z* score of ≤ − 3. Severe pneumonia was defined as history of cough or difficulty breathing plus lower chest wall indrawing, and very severe pneumonia was defined as cough or difficulty breathing plus either hypoxia, lethargy, loss of consciousness, prostration, or a history of convulsions.

A similar approach for surveillance in adults was established in January 2007. Blood cultures were taken for all patients presenting to the wards with a history of fever, axillary temperature of <36°C or >37.4°C, or signs of focal sepsis. Cerebrospinal fluid (CSF) cultures were taken in patients with any 2 of pyrexia (axillary temperature >37.4°C), meningism (neck stiffness and/or photophobia), or altered mental status.

A case was defined as a patient in whom *Salmonella enterica* species was cultured from blood or CSF.

### Procedures

Pediatric blood samples for bacterial cultures were collected in BACTEC Peds Plus bottles and processed with a BACTEC automated blood culture instrument (Becton Dickinson) for initial detection of bacteria in the blood. In adults, BACTEC Plus Aerobic/F bottles were used. BACTEC-positive samples were subcultured on standard media by routine microbiological techniques. Either biochemical test kits (API, bioMérieux), serological tests, or both were used to confirm suspected pathogens. Good Clinical Laboratory Practice was audited by Qualogy and external quality assurance was provided by the UK National External Quality Assessment service.

The following organisms were considered as contaminants: *Bacillus* species, coryneforms, *Micrococcus* species, coagulase-negative *Staphylococcus*, and viridans group *Streptococcus. Salmonella* serotypes were determined using the Kauffman–White scheme and commercial antisera. Antimicrobial susceptibilities were determined by broth dilution using Microscan panels (Siemens, Germany), and minimum inhibitory concentrations were interpreted using the latest Clinical and Laboratory Standards Institute guidelines [15]. Organisms that showed intermediate susceptibility results to any of the drugs tested were labeled as nonsusceptible to the drug. Multidrug resistance (MDR) was defined as nonsusceptibility to ≥2 of the following antibiotics: ampicillin, trimethoprim-sulfamethoxazole (cotrimoxazole), and tetracycline.

An automated counter (Beckman Coulter for children, Coulter ACT-5 for adults) measured blood counts and hemoglobin. Blood was examined for malaria parasites by Giemsa-stained thick and thin films at ×1000 magnification. Systematic HIV-1 testing was performed using rapid antibody tests according to national policy, which began in 2007 [16]. Prior to that, HIV testing was undertaken only in those seeking a result. DNA was extracted retrospectively from frozen samples collected at admission by use of Qiagen DNA blood mini-kits (Qiagen, Crawley, United Kingdom) and typed for sickle cell anemia by polymerase chain reaction.

### Statistical Analysis

Student *t* test or Wilcoxon rank-sum test were conducted for normal or nonnormally distributed continuous variables, respectively. We used logistic regression to establish the risk factors for NTS bacteremia compared to other admissions. Variables that were significant (*P* < .1) in the univariate analysis were included in the multivariable model. Odds ratios (ORs) and 95% confidence intervals (CIs) were reported.

Incidence was calculated among the residents of the KHDSS using the midyear population estimates as the denominator. Age-specific population estimates for the era before the KHDSS began were extrapolated using a log linear model of age-specific data based on subsequent enumerations. We adjusted the incidence to account for the proportion of patients who were eligible for blood cultures but missed them and for the proportion of cultures that were contaminated, assuming the distribution of true pathogens in contaminated vials was represented by the distribution in uncontaminated vials. In adults, we estimated the proportion of the KHDSS that was HIV infected using the population prevalence for Coast Province (4.3%) [17] and used this as a denominator in calculating the incidence of iNTS among HIV-infected individuals.

Hospital-based surveillance has been associated with underascertainment of cases illustrated by distance decay in the incidence of admissions to hospital in patients aged <5 years [18]. We adjusted the incidence of NTS for underascertainment in the number of patients presenting to KCH among children <5 years. We assumed that the true incidence of hospitalization with NTS infection in each location was proportional to the location-specific death rates in the KHDSS and that the location closest to the hospital (Kilifi Township) had complete ascertainment [18]. The analysis was performed using Stata 13 (StataCorp, College Station, Texas).

### Ethical Approval

We report results from a series of research studies that were approved by the National Ethical Review Committee of KEMRI (scientific steering committee 1067, 1357, and 1433). Patients were only investigated if we obtained informed written consent either from the patient or, in the case of children, from their parent or guardian.

## RESULTS

### Bacterial Isolation and Typing

**Figure 1.**
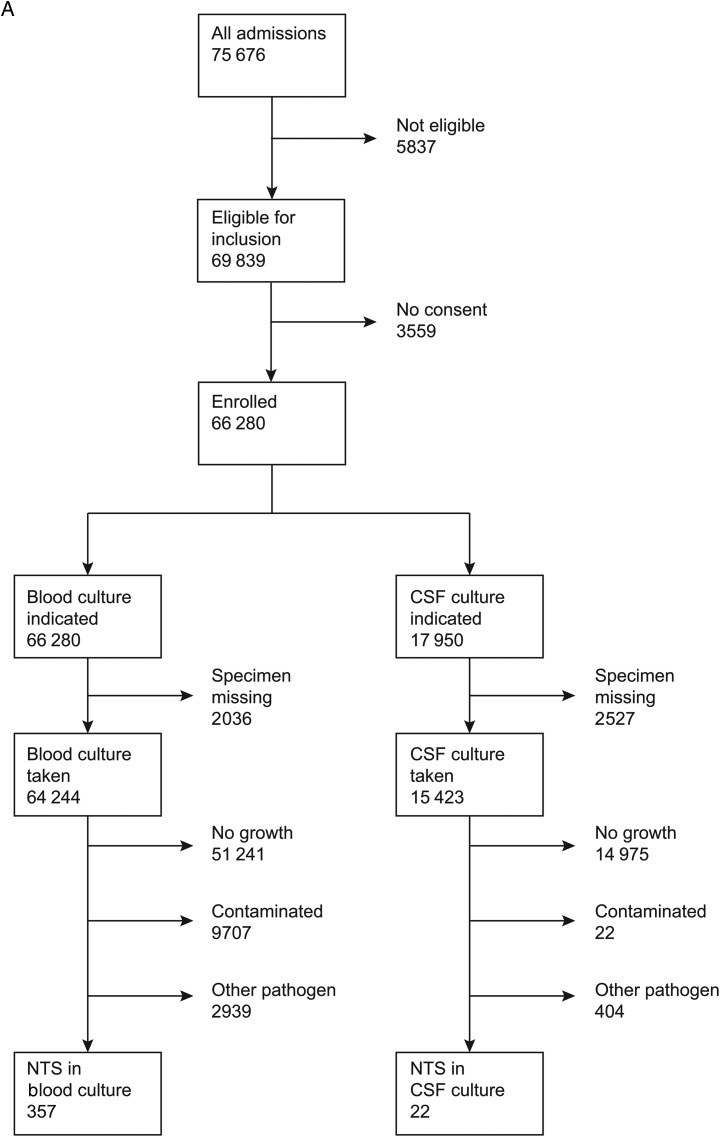

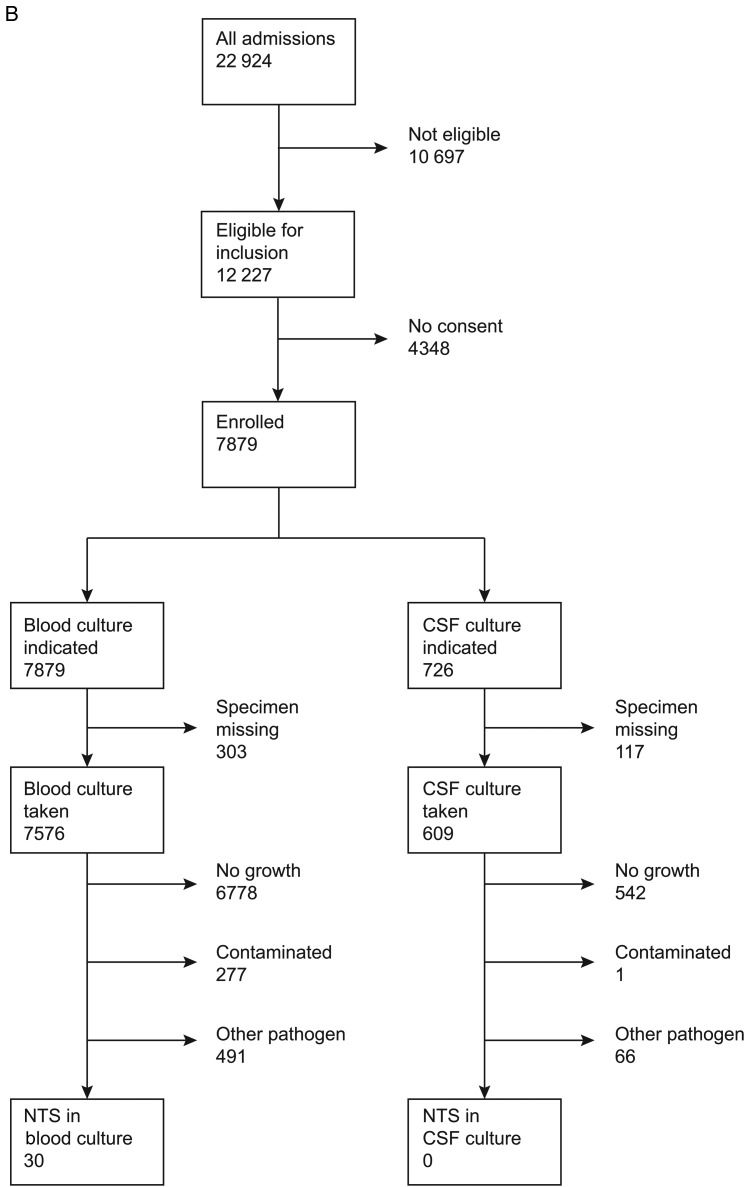
Consolidated Standards of Reporting Trials (CONSORT) diagram representing flow of patients. *A*, Children. *B*, Adults. Abbreviations: CSF, cerebrospinal fluid; NTS, nontyphoidal *Salmonella*.

Of 75 676 admissions among children, 3296 had bacteremia of which 357 (10.8%) were NTS and 15 (0.5%) were *Salmonella* Typhi isolates (Figure 1). Among 22 924 adult admissions, 521 had bacteremia, of which 30 (5.8%) were NTS and 11(2.1%) were *Salmonella* Typhi isolates. Of 387 isolates of NTS, 351 were serotyped; 160 (45.6%) were *Salmonella enterica* serovar Enteritidis, 152 (43.3%) were *Salmonella enterica* serovar Typhimurium, and 39 (11.1%) isolates could not be serotyped using the panel of antisera available. There were no *Salmonella* Paratyphi organisms isolated. There was 1 case of *Salmonella enterica* subspecies *arizonae* that was excluded from this analysis.

Twenty-two isolates of NTS were cultured from CSF samples taken on admission; 8 (36.4%) occurred in neonates <7 days old and all of them occurred in infants. Among these 22 infants, 19 (86.4%) had blood cultures positive for NTS. There were no cases of NTS meningitis in adults. *Salmonella* Typhi was not isolated from CSF in either children or adults.

Mixed infections with NTS were present in 10 patients, of whom 5 were coinfected with *S. pneumoniae*; the others had group A *Streptococcus* (3), *Escherichia coli* (1), and *Pseudomonas* species (1).

### Age and Sex Distribution

Among children and adults combined, the median age of infection with iNTS was 1 year (interquartile range [IQR], 0–3 years) and with *Salmonella* Typhi was 8.5 years (IQR, 2–18 years).

Among children, 199 of 357 (55.7%) patients with iNTS and 9 of 15 (60%) with *Salmonella* Typhi were male. The median age of children with iNTS was 15 months (IQR, 7–28 months) and with *Salmonella* Typhi was 34 months (IQR, 28–77 months).

Among adults, 11 of 30 (36.7%) patients with iNTS and 8 of 11 (72.7%) with *Salmonella* Typhi were male. The median age of infection with iNTS and with *Salmonella* Typhi was 30 years (IQR, 24–42 years) and 22 years (IQR, 18–39 years), respectively. The median age of infection with iNTS did not vary by sex. There were no differences in the serotype distribution across different age groups.

### Clinical Characteristics and Risk Factors for Invasive NTS Disease

Among children with iNTS, 261 of 351 (74.4%) presented with fever (axillary temperature >37.4°C), 99 of 352 (28.1%) had *Plasmodium falciparum* infection on their blood slide, 19 of 268 (7.1%) had sickle cell disease, and 60 of 249 (24.1%) were HIV infected. Significant risk factors were elevated temperature, malnutrition, splenomegaly, and HIV infection (Table 1).

**Table 1.** Characteristics of Patients Presenting With Invasive Nontyphoidal *Salmonella* Disease and Associated Risk Factors in Children and Adults

Characteristic	NTS Bacteremia, No. Positive/No. Tested (%)	Other Admissions, No. Positive/ No. Tested (%)	Unadjusted OR (95% CI)	*P* Value	Adjusted OR (95% CI)	*P* Value
Children	n = 357	n = 75 676				
Clinical features						
Sex, male	199 (55.7)	42 495/75 676 (56.4)	0.96 (.78–1.17)	.667		
Temperature						
Low	24/351 (6.9)	11 070/74 361 (14.9)	0.78 (.49–1.21)	.271	0.24 (.07–.80)	.020
Normal	66/351 (18.8)	22 792/74 361 (30.7)				
High	261/351 (74.4)	40 499/74 361 (54.5)	2.17 (1.66–2.82)	<.001	2.03 (1.27–3.28)	<.001
Diarrhea	103/344 (29.9)	13 739/72 561 (18.9)	1.88 (1.50–2.36)	<.001		
Vomiting	110 (32.0)	19 121/73 396 (26.1)	1.33 (1.06–1.67)	.012		
Malnutrition	103 (31.4)	12 619/70 584 (17.9)	2.16 (1.71–2.71)	<.001	2.25 (1.47–3.44)	<.001
Severe pneumonia	61 (17.1)	12 563/75 676 (16.8)				
Very severe	60 (16.9)	10 324/75 676 (13.8)	1.2 (.84–1.71)	.319		
Pneumonia						
Splenomegaly	40 (19.0)	3351/56 026 (6.0)	3.66 (2.60–5.15)	<.001	4.61 (2.70–7.86)	<.001
Hepatomegaly	37 (17.6)	4133/56 027 (7.4)	2.67 (1.88–3.79)	<.001		
Laboratory features						
Malaria slide positive	99/352 (28.1)	18 645/71 973 (25.9)	1.08 (.85–1.36)	.532		
HIV positive	60/249 (24.1)	1998/31 077 (6.4)	4.53 (3.39–6.06)	<.001	5.16 (3.29–8.07)	<.001
Adults	n = 30	n = 22 894				
Clinical features						
Sex, male	11 (36.7)	9846/22 894 (43.0)	0.77 (.36–1.61)	.485		
Temperature						
Low	9 (30.0)	7336/21 339 (34.4)	1.37 (.54–3.46)	.501		
Normal	9 (30.0)	10 076/21 339 (47.2)	1			
High	12 (40.0)	3927/21 339 (18.4)	3.42 (1.44–8.13)	.005		
Diarrhea	14 (46.7)	1604/21 541 (7.4)	10.88 (5.30–22.32)	<.001	4.34 (1.88–10.02)	.001
Vomiting	12 (40.0)	2501/21 542 (11.6)	5.08 (2.44–10.55)	<.001		
Splenomegaly	2 (6.7)	217/21 527 (1.0)	7.01 (1.66–29.63)	.008		
Hepatomegaly	5 (16.7)	762/21 528 (3.5)	5.45 (2.08–14.28)	.001		
Laboratory features						
Malaria slide positive	0/24 (0)	251/7771 (3.2)	1.00 (1.00–1.00)			
HIV positive	21/23 (91.3)	2583/9351 (27.6)	27.51 (6.45–117.42)	<.001	20.41 (4.67–89.21)	<.001

Abbreviations: CI, confidence interval; HIV, human immunodeficiency virus; NTS, nontyphoidal *Salmonella*; OR, odds ratio.

Among 30 adults with NTS bacteremia, only 12 (40.0%) presented with a febrile illness and none were infected with *P. falciparum*. HIV infection was present in 21 of the 23 (91.3%) patients tested. In the final model, significant risk factors for iNTS infection were HIV (OR, 20.4 [95% CI, 4.67–89.21]) and diarrhea (OR, 4.34 [95% CI, 1.88–10.02]).

Recurrent infection was observed in only 1 female HIV-infected adult admitted with NTS bacteremia, 6 months after the first admission. The serotype isolated was *Salmonella* Typhimurium in both admissions.

### Incidence

**Figure 2.**
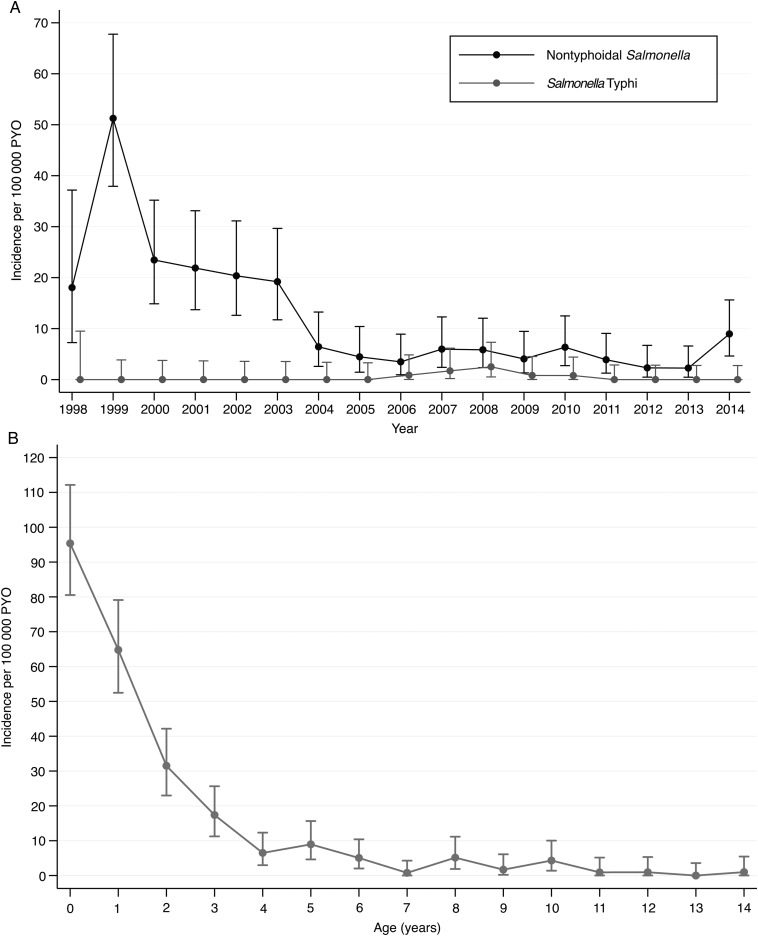
*A*, Crude incidence (and 95% confidence intervals [CIs]) of nontyphoidal (NTS) bacteremia among children (<15 years) across the study period. *B*, Crude age-incidence curve (and 95% CIs) of NTS bacteremia among children.

There were 219 cases of iNTS and 13 cases of *Salmonella* Typhi occurring in residents of the KHDSS, representing 57% and 50% of all hospitalized cases, respectively. The incidence of iNTS among children aged <5 years was 25.6 per 100 000 person-years of observation (PYO). The rate adjusted for missing specimens and contaminated cultures was 32.6 (95% CI, 28.1–37.7) and the rate adjusted, in addition, for access to care was 36.4 per 100 000 PYO (Table 2). The incidence varied by age (Figure 2*B*) but was highest among newborn infants <7 days old at 174.4 per 100 000 PYO (crude incidence, 136.6 per 100 000 PYO). Variation across the locations of the KHDSS was marked (range, 4.8–69.8 per 100 000 PYO), with the highest rates from locations south of KCH (Figure 2*C*). By Poisson regression, the incidence of iNTS among children aged <5 years declined, on average, by 16% in each year of the study period (incidence rate ratio [IRR], 0.84 [95% CI, .81–.86]) whereas the incidence of *Salmonella* Typhi bacteremia remained constant (IRR, 1.05 [95% CI, .90–1.22]). In children, the incidence of *Salmonella* Typhimurium and *Salmonella* Enteritidis in children declined by 14% (IRR, 0.86 [95% CI, .81–.90]) and 21% (IRR, 0.79 [95% CI, .74–.83]) per year, respectively (Supplementary Figure 1).

**Table 2.** Crude and Adjusted Incidence Rates and Case Fatality Rate of Invasive Nontyphoidal *Salmonella* Disease Across Different Age Groups

Age Group, y	Cases	Deaths	CFR (%)	Cases in KHDSS	Person-years of Observation	Crude Incidence (95% CI)	Adjusted Incidence^a^ (95% CI)	Access-Adjusted Incidence (95% CI)
0–4	321	71	22	186	726 881	25.6 (22.0–29.5)	32.6 (28.1–37.7)	36.4 (35.6–37.1)
5–14	36	3	8.3	22	1 174 129	1.9 (1.17–2.84)	2.4 (1.49–3.62)	…
≥15	30	11	37	11	1 056 038	1.0 (.52–1.86)	1.7 (.87–3.11)	…

Incidence rates are given per 100 000 person-years.

Abbreviations: CFR, case fatality rate; CI, confidence interval; KHDSS, Kilifi Health and Demographic Surveillance System.

^a^ Adjustment based on missed blood cultures and contamination rate per age group.

**Figure:**
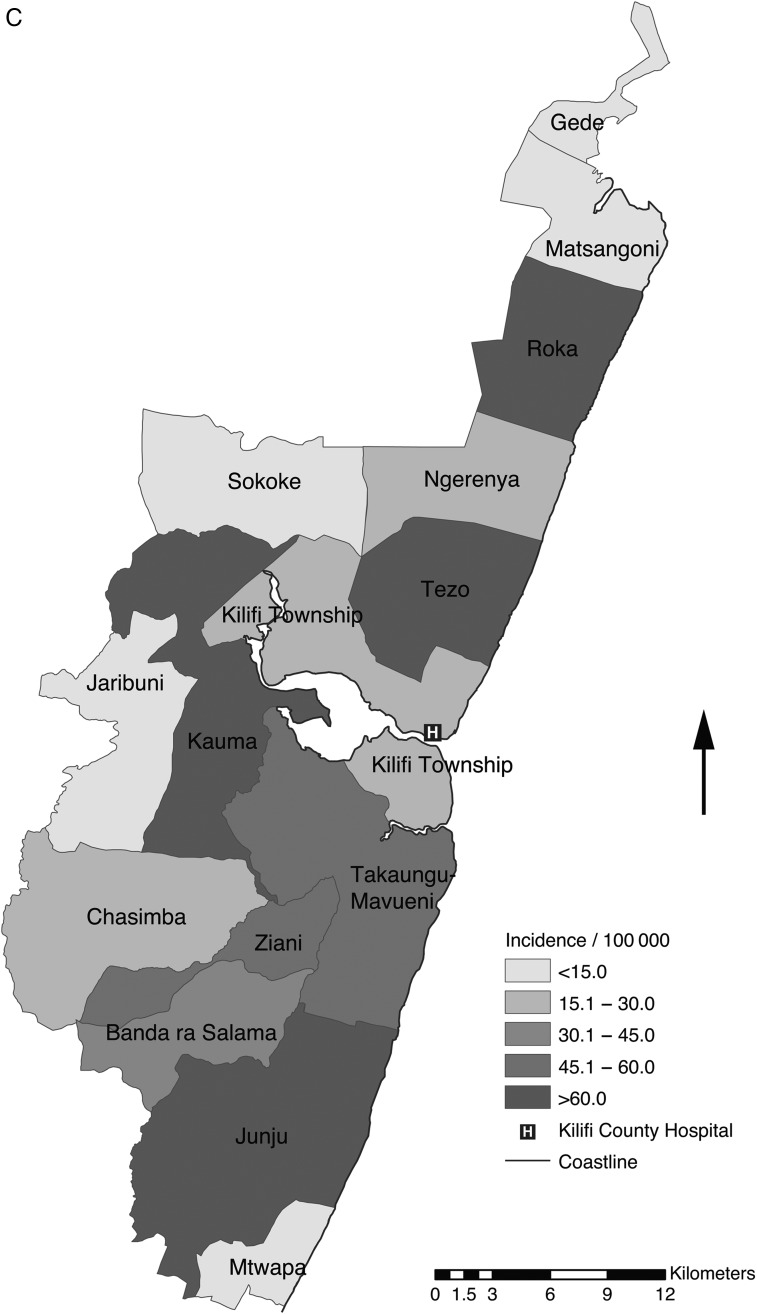
*Figure 2 continued*. *C*, Map of the Kilifi Health and Demographic Surveillance System showing geographical distribution of the access-adjusted rates of NTS bacteremia among children aged <5 years. Abbreviation: PYO, person-years of observation.

In adults, the adjusted incidence of iNTS was 1.7 per 100 000 PYO. Among HIV-infected individuals, the crude incidence of iNTS was 13.2 per 100 000 PYO compared with a crude incidence of 0.1 per 100 000 PYO among HIV-uninfected individuals (IRR, 133.5 [95% CIs, 16.2–6142.4]). The incidence of iNTS in adults did not vary significantly by time. For *Salmonella* Typhi infections, the incidence among children and adults was 0.4 and 0.5 per 100 000 PYO, respectively.

### Antimicrobial Susceptibility

Among 351 NTS isolates, in vitro resistance to ampicillin, trimethoprim-sulfamethoxazole, tetracycline, and cefotaxime was observed in 98 (27.9%), 86 (24.5%), 35 (10.0%), and 21 (6.0%), respectively (Supplementary Table 1). Eighty-four (23.9%) of the NTS isolates were MDR. Of the 21 patients showing nonsusceptibility to third-generation cephalosporins, 8 (38.1%) died during the admission.

**Figure 3.**
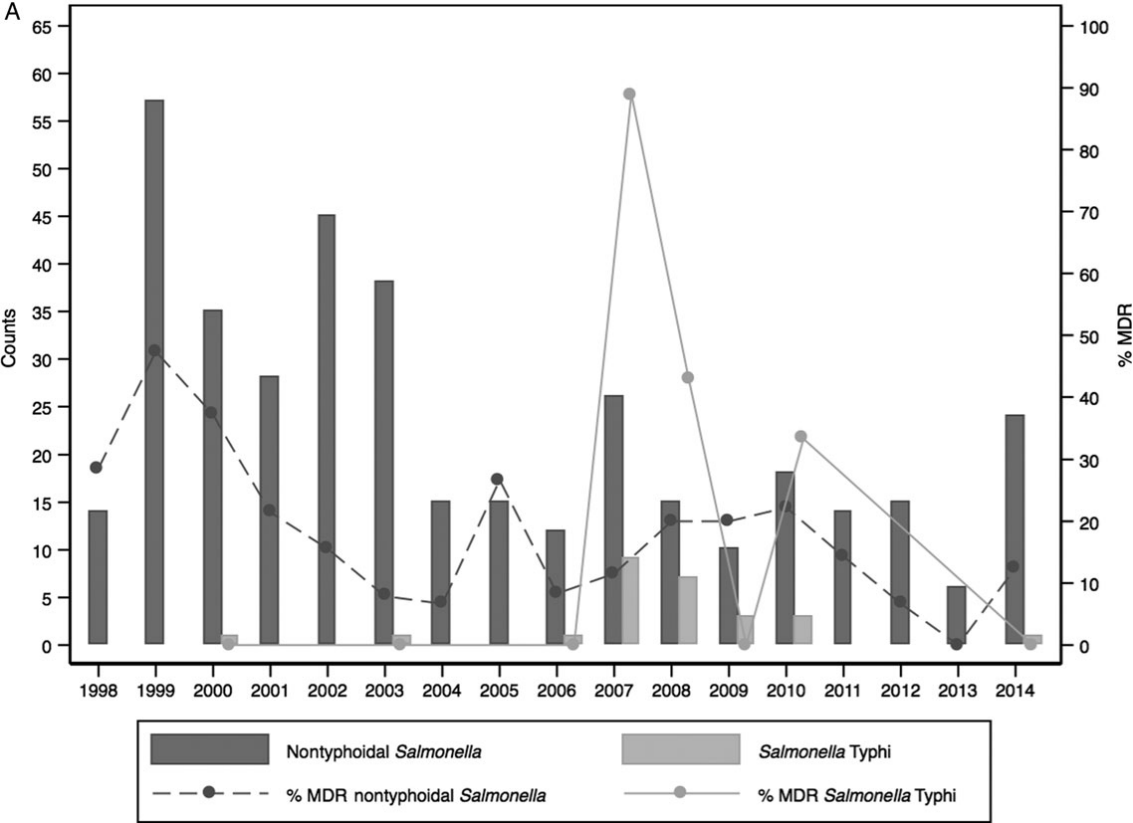

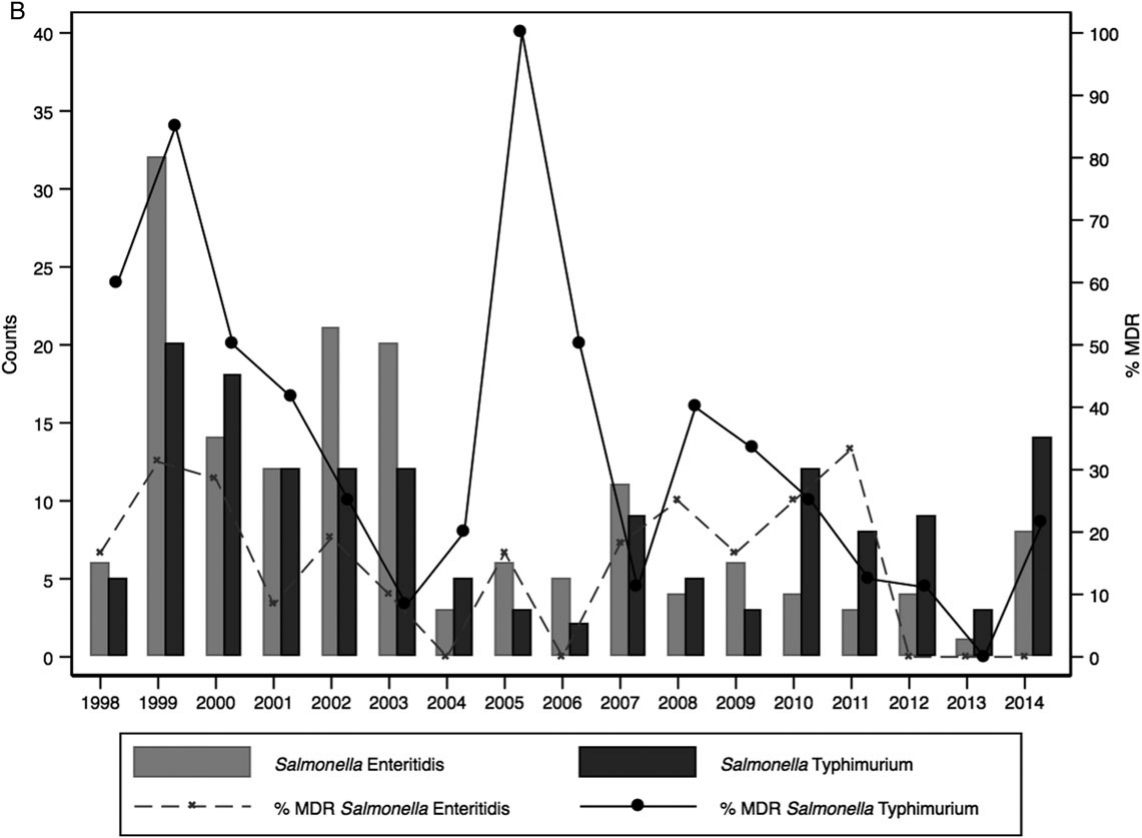
The relationship between multidrug resistance (MDR) and the number of nontyphoidal *Salmonella* (NTS) and *Salmonella* Typhi (*A*) or the number of NTS isolates per serotype (*B*) among children, August 1998–2014.

Among the NTS serotypes, resistance to individual drugs was mainly driven by *Salmonella* Typhimurium, which also had a higher percentage of MDR isolates (55/152 [36.2%]). Twenty of the 21 isolates showing nonsusceptibility to third-generation cephalosporins were *Salmonella* Typhimurium. Multidrug resistance in iNTS serotypes showed temporal variability with several peaks across the years. A peak in MDR *Salmonella* Typhimurium in 2005 was followed sequentially by a shift in dominance over *Salmonella* Enteritidis (Figure 3). Peaks in MDR isolates of *Salmonella* Enteritidis were seen over time with no apparent change in the proportion of isolates in subsequent years.

Of *Salmonella* Typhi, 12 of 26 (46.2%) were MDR. Eight (66.7%) of these MDR isolates were isolated in 1 year (2007). *Salmonella* Typhi isolates were all susceptible to cefotaxime.

### Case Fatality Ratio (CFR)

Among 321 cases of iNTS disease in children aged <5 years, 71 (22.1%) died in hospital (Table 2). The CFR for newborn infants <7 days old was 57.1% (4/7) and for NTS meningitis was 45.5% (10/22). Among 10 children with mixed infections of NTS and another pathogen, 6 died in hospital. Among adult cases of iNTS, 11 of 30 (36.7%) died in hospital. Among 26 patients of all ages with *Salmonella* Typhi bacteremia, 1 (3.8%) adult died.

## DISCUSSION

We describe the characteristics of 387 cases of iNTS disease and 26 cases of *Salmonella* Typhi diagnosed in a rural hospital in Kenya. The importance of NTS as a cause of bacteremia in children has been previously described in this setting and other countries in Africa [5, 6, 10, 19]. Newborns and infants are those most susceptible to iNTS [5, 20] and in our study had the highest CFRs; more than half of all infected newborn infants died during the hospital admission.

Our results show a decrease in the incidence of iNTS over time in children. This is most likely associated with the decline in malaria infections over the same period [21]. The association of NTS with recent or current malaria has been repeatedly documented in children [22–24]. In our analysis, current malaria infection was not significantly associated with iNTS infection but splenomegaly, a marker of recent malaria infection, was. Malnutrition and HIV infection were independent risk factors of iNTS infection.

In previous studies, sickle cell anemia has been shown to be strongly associated with iNTS disease [25]. Among our pediatric cases with iNTS, the prevalence of sickle cell anemia was 7.1% compared with a prevalence of 1% in a birth cohort of >10 000 children recruited during the same period in Kilifi and investigated in infancy [21]. None of the cases of *Salmonella* Typhi tested had sickle cell anemia.

Among adults, HIV was the principal risk factor for iNTS disease. The incidence of iNTS among HIV-infected patients was 133 times higher than among HIV-uninfected individuals. In an area with a HIV prevalence of 4.3%, this is highly significant. Among adults, iNTS disease was more common in females than in males, reflecting the higher prevalence in women (6.1%) than in men (2.6%) in Kenya [17]. None of the adult cases had a positive malaria slide, although the adult surveillance was only established in 2007 when the prevalence of malaria in Kilifi had already fallen substantially [21].

Only 1 adult case of *Salmonella* Typhi infection was HIV infected. Similar low prevalence of HIV in cases of *Salmonella* Typhi has been shown in studies from Tanzania [26, 27]. The incidence of *Salmonella* Typhi across all age groups was 0.5 per 100 000 PYO, which yielded insufficient cases in which to examine risk factors. In addition, the CFR for typhoid fever in hospital was low at 1 in 26 (3.8%). Although the majority of the *Salmonella* Typhi cases reported here were in children (58%), there were no deaths in children. Culture-confirmed *Salmonella* Typhi infections in Africa are less common than NTS infections but are especially associated with urban areas with poor hygiene and sanitation [2, 6]. *Salmonella* Typhi infections commonly occur in epidemics [28, 29]. In our study, we retrospectively identified a small outbreak of MDR *Salmonella* Typhi that occurred in 2007–2008 that was associated with 1 death.

Previous reports from Kilifi have shown higher susceptibility to amoxicillin and cotrimoxazole among iNTS isolates compared with *Escherichia coli* and other gram-negative bacilli by Etest [30]. This general susceptibility to common antibiotics was seen in our study, with the prevalence of MDR declining over time. The majority of the MDR isolates were *Salmonella* Typhimurium. These could be the distinct genotype ST313 that has been identified among MDR *Salmonella* Typhimurium causing invasive disease in Kenya and Malawi [31]; unfortunately, because we were unable to do multilocus sequence typing, this cannot be confirmed. The presence of resistance to third-generation cephalosporins among iNTS is alarming and we intend to characterize these isolates further. In low-income countries, extended MDR NTS infections have limited options for alternative therapy, especially among those who are HIV infected [32], and are associated with high mortality.

With incomplete access to care and low blood culture sensitivity for iNTS, the incidence at population level is difficult to measure and is often underestimated as a consequence. We show a marked variation in the incidence by location, with the locations further from KCH having the lowest incidence. However, after adjusting for access to care, the incidence estimate for iNTS admissions among <5-year-olds increased by only 12%, suggesting that for a disease as severe and potentially fatal as iNTS, the barriers to hospital admission are comparatively small.

We have reported one of the longest series of continuous surveillance for invasive bacterial disease in sub-Saharan Africa using consistent demographic, clinical, and laboratory methods. In a rural setting with a high burden of malaria initially and a low prevalence of HIV infection, we have observed a high incidence of iNTS disease among children and a low incidence of typhoid fever. The burden of iNTS in children has declined in synchrony with the dramatic reduction in malaria parasitemia in the study area. However, over the whole period it was the second commonest cause of invasive bacterial infection and had a CFR of 23% in children <5 years of age. It is an especially fatal disease in newborn infants. Theories linking malaria to iNTS disease in children do not readily explain the high risk of disease in neonates who do not often get malaria. As pneumococcal disease is brought under control by vaccination, invasive NTS disease may become the most important cause of sickness and death due to invasive bacterial infections in children in Kilifi, and a common disease of adults. Public health measures to control NTS disease and its transmission, possibly including effective vaccination, are likely to improve child and adult health in the region.

## Supplementary Data

Supplementary materials are available at *Clinical Infectious Diseases* online (http://cid.oxfordjournals.org). Supplementary materials consist of data provided by the author that are published to benefit the reader. The posted materials are not copyedited. The contents of all supplementary data are the sole responsibility of the authors. Questions or messages regarding errors should be addressed to the author.

Supplementary Data
