# Supplementary material for: Invasive Salmonellosis in Kilifi, Kenya
Source: Clin Infect Dis. 2015 Oct 7;61(Suppl 4):S290–301. doi: 10.1093/cid/civ737 (PMC4596936; doi:10.1093/cid/civ737)
Supplement: Supplementary Data [file supp_61_suppl-4_S290__index.html]

Supplementary Data 

# Invasive Salmonellosis in Kilifi, Kenya

## Supplementary Data

Supplementary Data

- Supplementary Data - Docx file
