# Supplementary material for: Invasive Salmonellosis in Kilifi, Kenya
Source: Clin Infect Dis. 2015 Oct 7;61(Suppl 4):S290–301. doi: 10.1093/cid/civ737 (PMC4596936; doi:10.1093/cid/civ737)
Supplement: Supplementary Data [file supp_civ737_civ737supp.docx]

**Supplementary material**

**Figure S1.** Crude incidence (and 95% confidence intervals) of *Salmonella* Typhimurium and *Salmonella* Enteritidis bacteremia in children (under 15 years) across the study period.

**
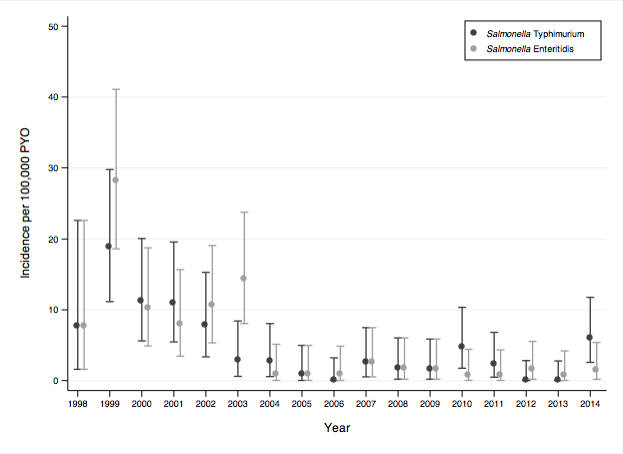
**

**Figure S2:** Non-susceptibilityprofile of individual antimicrobial drugs against iNTS isolates across the study period (% Resistant).


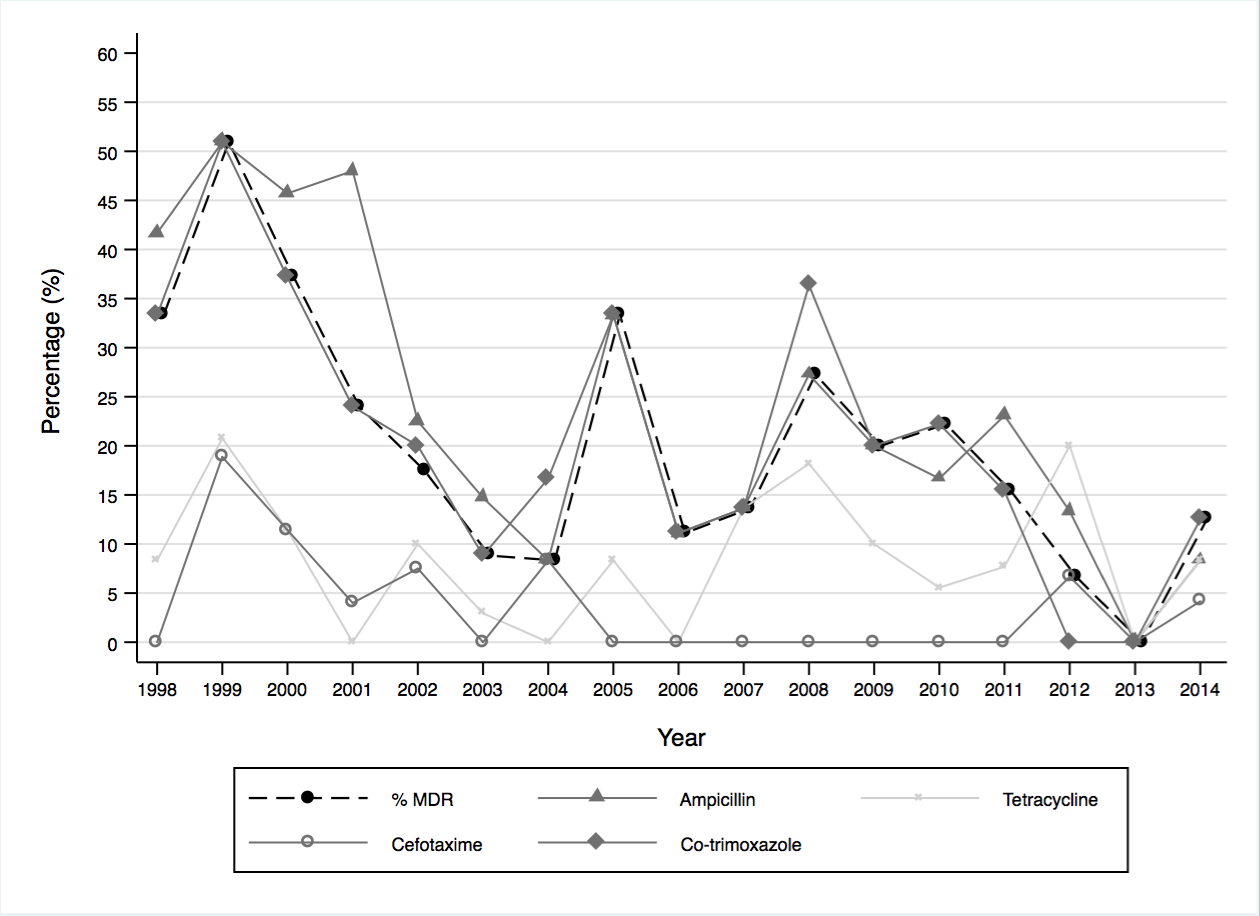


**Table S1:** Susceptibility of individual drugs to *Salmonella* Enteritidis,*Salmonella* Typhimurium, non-typhoidal *Salmonella* and*Salmonella*Typhi by MIC (% Susceptible)

| **Antibiotic** | ***S.* Enteritidis**  **N=159** | | ***S.* Typhimurium**  **N=152** | | **iNTS**  **N=351** | | ***S.* Typhi**  **N=26** | |
| --- | --- | --- | --- | --- | --- | --- | --- | --- |
|  | n | n/N(%) | n | n/N(%) | n | n/N(%) | n | n/N(%) |
| Ampicillin | 128 | 80.5 | 85 | 55.9 | 253 | 72.1 | 13 | 50.0 |
| Ampicillin-Sulbactam | 128 | 80.5 | 86 | 56.6 | 253 | 72.1 | 15 | 57.7 |
| Cefotaxime | 159 | 100 | 132 | 86.8 | 330 | 94.0 | 26 | 100 |
| Co-trimoxazole | 128 | 80.5 | 97 | 63.8 | 265 | 75.5 | 14 | 53.9 |
| Tetracycline | 134 | 84.3 | 142 | 93.4 | 316 | 90.0 | 14 | 53.9 |

n represents the number susceptible
